# Maternal prenatal anxiety and child *COMT* genotype predict working memory and symptoms of ADHD

**DOI:** 10.1371/journal.pone.0177506

**Published:** 2017-06-14

**Authors:** Kieran J. O’Donnell, Vivette Glover, Jari Lahti, Marius Lahti, Rachel D. Edgar, Katri Räikkönen, Thomas G. O’Connor

**Affiliations:** 1 Ludmer Centre for Neuroinformatics and Mental Health, Douglas Hospital Research Centre, Department of Psychiatry, McGill University, Montreal, Canada; 2 Child and Brain Development Program, Canadian Institute for Advanced Research, Toronto, Canada; 3 Institute of Reproductive and Developmental Biology, Imperial College London, United Kingdom; 4 Department of Psychology and Logopedics, Faculty of Medicine, University of Helsinki, Finland; 5 Helsinki Collegium for Advanced Studies, University of Helsinki, Finland; 6 Queen's Medical Research Institute, University of Edinburgh, United Kingdom; 7 Centre for Molecular Medicine and Therapeutics, University of British Columbia, Vancouver, Canada; 8 Wynne Center for Family Research, Department of Psychiatry, University of Rochester Medical Center, Rochester, New York, United States of America; Universite de Rennes 1, FRANCE

## Abstract

Maternal prenatal anxiety is an important risk factor for altered child neurodevelopment but there is uncertainty concerning the biological mechanisms involved and sources of individual differences in children’s responses. We sought to determine the role of functional genetic variation in *COMT*, which encodes catechol-O-methyltransferase, in the association between maternal prenatal anxiety and child symptoms of ADHD and working memory. We used the prospectively-designed ALSPAC cohort (n = 6,969) for our primary data analyses followed by replication analyses in the PREDO cohort (n = 425). Maternal prenatal anxiety was based on self-report measures; child symptoms of ADHD were collected from 4–15 years of age; working memory was assessed from in-person testing at age 8 years; and genetic variation in *COMT* at rs4680 was determined in both mothers and children. The association between maternal prenatal anxiety and child attention/hyperactivity symptoms and working memory was moderated by the child’s rs4680 genotype, with stronger effects obtained for the val/val (G:G) genotype relative to val/met (A:G) (all p<0.01) and met/met (A:A) groups (all p<0.05). Similar findings were observed in the PREDO cohort where maternal prenatal anxiety interacted with child rs4680 to predict symptoms of ADHD at 3.5 years of age. The findings, from two cohorts, show a robust gene-environment interaction, which may contribute to inter-individual differences in the effects of maternal prenatal anxiety on developmental outcomes from childhood to mid-adolescence.

## Introduction

There is now a sizable evidence base from research groups in several countries linking psychological distress in pregnancy with altered neurodevelopment—cognitive and intellectual abilities, executive functions, language, and brain imaging—in the children, adolescents and young adults [1–10]. These findings provide leads in identifying mechanisms of brain development, highlight the significance of prenatal exposures for child neurodevelopment, and expand the public health implications of maternal prenatal distress for the mother and child. The current study extends this research by investigating sources of individual differences in the link between prenatal maternal anxiety and child neurodevelopment.

Genetic variation is a likely source of differences in response to prenatal maternal mood; a focus on *COMT* is particularly relevant for research on neurodevelopment. A functional single nucleotide polymorphism (SNP) within *COMT* (val158met; rs4680) results in a valine to methionine mutation and is strongly related to COMT enzyme activity. Compared with the val allele, the met allele is associated with significantly lower enzymatic activity. This is of particular importance in brain regions such as the prefrontal cortex in which COMT is one of the principal regulators of extracellular dopamine levels, resulting in higher levels of dopamine and lower dopamine turnover in met homozygotes (A:A genotype) compared with carriers of the val/val (G:G genotype)[11]. Numerous studies report that individuals with one or two copies of the val allele exhibit poorer performance on neurodevelopmental assessments in adults [12] and children [13] and exhibit greater vulnerability for psychiatric disorder associated with deficits in neurodevelopment [14]. Furthermore, Knickmeyer and colleagues reported reduced volume in the temporal cortex associated with the val/val homozygote in their study of newborns [15].

Relevant to the current study is evidence that the well-documented association between prenatal maternal anxiety or stress and child outcome cited above may be moderated by the child’s *COMT* genotype [16]. A report from Singapore on 146 neonates indicated that the association between prenatal maternal anxiety and neonatal cortical morphometry was moderated by genetic variation in *COMT* [16], with increased thickness of the ventrolateral prefrontal cortex observed in neonates with the val allele. This finding is notable in suggesting that *COMT* may be involved in early brain development and, therefore, relevant for studies of prenatal anxiety. The finding is also notable for extending the focus in prenatal maternal anxiety and stress research beyond glucocorticoids to also include dopamine-mediated mechanisms. On the other hand, it is not yet clear if *COMT* moderation of prenatal maternal anxiety represents a robust effect, on other child outcomes. A report of 546 children using a measure of maternal perceived stress at one-day postpartum suggested that child rs4680 may moderate prenatal exposure [17], with poorer outcomes on a composite measure of total behavioral and emotional problems observed in met/met homozygotes. Given the potential significance of these findings for identifying alternative biological mechanisms, replication is warranted and extension is needed to other measures of neurodevelopment and to assessments beyond infancy.

In the current study we describe the effects of maternal prenatal anxiety on two key markers of neurodevelopment in the child: a) working memory as assessed from in-person standardized testing and b) attention problems from measures at 4 and 15 years of age. We examine child rs4680 as a main effect and moderator of the prenatal anxiety exposure. The longitudinal assessment of symptoms of inattention and hyperactivity from preschool through mid-adolescence available in the current study with the ALSPAC cohort, provided a rare opportunity to examine genetic moderation of the prenatal prediction across a period of dramatic change in neurodevelopment. Based on our previous research [4, 18] and the limited research cited above, we hypothesized that variation at rs4680 would moderate the association between maternal prenatal anxiety and ADHD-related child outcomes. We test this hypothesis in a large prospective longitudinal cohort and a smaller independent replication sample. We also apply stringent controls for type 1 errors associated with gene-environment analyses [19].

## Materials and methods

### Primary study sample

Data for this study are part of the Avon Longitudinal Study of Parents and Children (ALSPAC), an ongoing population-based study designed to investigate the effects of a wide range of influences on the health and development of children [20]; details available at http://www.bris.ac.uk/alspac. The study cohort consisted of 14,541 pregnancies and 13,971 children who were still alive at 12 months of age. The current analyses focus on mothers and their child (n = 6,969; see inclusion criteria below) who provided biological samples for genetic analyses, questionnaire data on their child’s emotional and behavioral development at 4 years of age and a subset of these who returned at age 8 and 15 years for an in-person assessment in the research clinic. Written informed consent was obtained from parents, while children provided assent after receiving a full explanation of the study; ethical approval for all measures was obtained from the ALSPAC Ethics and Law Committee and from Local Research Ethics Committees.

### Measures

#### Maternal anxiety and depressive symptoms in pregnancy and the postnatal period

Maternal prenatal anxiety was based on the anxiety subscale of the Crown-Crisp experiential index (CCEI) and the Spielberger Trait Anxiety Inventory (STAI), both well-validated self-rating questionnaires with good disriminant validity and internal consistency for the CCEI (Cronbach’s α = 0.74; [21, 22]) and STAI (Cronbach’s α ≥ 0.86; [23]). Data were collected at 18 and 32 weeks gestation and at 8 weeks and 33 months postpartum (CCEI), and at 97 months postpartum (STAI). In our analyses we include a measure of maternal anxiety at 8 weeks postnatal (to account for particular effects in the immediate postnatal period), at 33 months (the point closest in time to the first assessment of child symptoms of ADHD) or at 97 months (the point closest in time to the assessment of adolescent symptoms); see data analysis section. Although our primary interest is in prediction from prenatal anxiety based on previous literature (e.g., [16]), we also consider maternal depressive symptoms based on maternal self-reports on the Edinburgh postnatal depression scale (EPDS), a ten item questionnaire shown to be valid both in and outside of the postnatal period [24, 25]. We include depression in analyses to examine the specificity of the interaction effect between maternal prenatal mood disturbance and child genotype on child health outcomes given that maternal depression, anxiety and stress may have differential effects on child outcomes [26].

Paternal symptoms of anxiety and depression were assessed using the CCEI and EPDS at one time point during pregnancy (18 weeks gestational age) and from 8 weeks to 33 months postpartum. We incorporate measures of paternal mood to examine the specificity of the interaction between measures of parental mood in the prenatal period and child *COMT* genotype. If the effects of maternal prenatal mood on child outcome are moderated by child *COMT* genotype, and these effects are specific to events occuring *in utero*, then we would expect a stronger prediction from maternal rather than paternal prenatal anxiety.

#### Child attention problems

Child ADHD symptoms at age 4 years based on maternal report were assessed using the Strengths and Difficulties questionnaire (SDQ), a widely used and well-validated assessment of child emotional and behavioral problems [27]. When the children were approximately 15 years of age, mothers provided information on child ADHD symptoms using the Development and Well-Being Assessment (DAWBA), a structured interview with considerable evidence for its clinical validity and reliability [28]. The DAWBA data are presented in probability bands that assess the likely prevalence of ADHD in one of six bands, e.g., Band 1 (<0.1%) = <0.1% of children in this band have the disorder in question, Band 2 (~0.5%) = ~0.5% of children in this band have the disorder in question, through to Band 6 (>70%) in which >70% of children in this band are likely to have the disorder in question [29].

#### General cognitive ability and working memory

Child backward digit span task [30] from the the Weschler Intelligence Scale for Children (WISC-III) [31] was assessed as part of an in-person standardized assessment of cognitive ability at 8 years of age. We focus our analyses on the backward digit span as a measure of working memory linked to central executive function (see [32, 33]). We also include in our analyses forward digit span and overall IQ.

#### *COMT* rs4680 genotype

Genotyping methods used within ALSPAC have been described previously [34] and *COMT* genetic methods have been previously reported [35]. DNA was obtained from whole blood samples. *COMT* rs4680 was determined using a 5′-nuclease fluorescence assay; primer/probe combinations were designed using Primer Express software (2.0) and allele discrimination performed using the Taqman 7900 PCR system (ABI, Foster City, Calif.). Allelic frequencies are provided in Table 1. Paired maternal rs4680 data were available on a subsample of this cohort (n = 4,590: AA n = 1214, AG n = 2296, GG n = 1080).

**Table 1:** Maternal mood, child and adolescent outcomes across rs4680 genotypes.

	*COMT* rs4680		
Maternal mood	AA (Met/Met)	GA(Val/Met)	GG (Val/Val)
	Mean (SD)	Mean (SD)	Mean (SD)
Prenatal anxiety (32 wks)	4.88 (3.48)	4.90 (3.52)	4.93 (3.45)
n	1888	3426	1655
Postnatal anxiety (8 wks)	3.29 (3.31)	3.29 (3.17)	3.29 (3.22)
n	1832	3348	1598
Postnatal depression (8 wks)	5.79 (4.66)	5.86 (4.61)	5.88 (4.65)
n	1858	3378	1619
Maternal anxiety (33 mths)	4.55 (3.46)	4.56 (3.52)	4.58 (3.45)
n	1636	2984	1422
Maternal depression (33 mths)	6.12 (4.94)	6.00 (4.97)	6.12 (4.85)
n	1640	2984	1420
Maternal anxiety (8 yrs)	39.37 (9.18)	39.19 (9.05)	38.92 (8.76)
n	1297	2399	1134
**Child outcomes**			
ADHD symptoms (SDQ) (4 yrs)	3.96 (2.33)	3.88 (2.32)	3.97 (2.32)
n	1583	2909	1415
Total IQ (WISC) (8 yrs)	105.87 (16.18)*	104.77 (16.21)	104.26 (15.88)
n	1341	2491	1193
Forwards digit span (8 yrs)	5.30 (1.08)	5.28 (1.07)	5.27 (1.12)
Backward digit span (8 yrs)	3.55 (0.81)	3.52 (0.84)	3.54 (0.85)
DAWBA Bands (15 yrs) %per group with likely ADHD	**AA (Met/Met) n =**	**GA(Val/Met) n =**	**GG (Val/Val) n =**
<0.1%	672	1187	572
~0.5%	133	253	109
~3%	50	101	64
~15%	24	56	33
~50%	4	7	3
>70%	1	7	3

Table presents descriptive statistics for measures of maternal mood, child and adolescent outcomes. Between-group differences were tested using one-way ANOVA and Χ^2^ tests where appropriate. Percent Likelihood of ADHD per group at 15 years is based on the Development and Well-Being Assessment. SDQ = Strengths and Difficulties Questionnaire. WISC = Wechsler Intelligence Scale for Children.

*One-way ANOVA *F*_*(2*, *5022)*_ = 3.44, p = 0.03 Fischer’s least significant difference AA vs AG p = 0.04, AA vs GG p = 0.01. wks = weeks, mths = months, yrs = years.

Our focus is on *COMT* (rs4680) because it has a clear link with neurodevelopment and has been investigated in prior work on genetic moderation, but we also include, for exploratory purposes, analyses of *BDNF* (rs11030121 and rs7124442), which we have previously shown to moderate maternal prenatal anxiety in predicting child emotional problems [36].

#### Obstetric and psychosocial covariates

Several possible covariates indexing socio-economic, demographic, and psychosocial risk were considered. Maternal education (highest educational achievement within the UK educational system according to 4 categories ranging from Certificate of Secondary Education/vocational training to university degree or higher degree) and household crowding (a 4-point scale created by dividing the number of people in the household by the number of rooms: 0–0.50, 0.50–0.75, 0.75–1.00 and > 1.00) at time of pregnancy were used as measures of maternal socioeconomic status. Data on prenatal risk factors such as maternal age, smoking and alcohol/substance abuse were collected during pregnancy. Birthweight and gestational age were recorded from medical notes. We did not exclude any participants based on birthweight or preterm birth however both obstetric outcomes were included as covariates in all analyses. At 24 months mothers completed a series of questions related to the frequency of positive (e.g., playing, cuddling, eating with, or praising child) and negative (e.g., slapping and shouting) parenting behaviors, which were summed to calculate a parenting score ranging from 18 (low) to 40 (high) [37]. The ALSPAC study website contains details of all the data that is available through a fully searchable data dictionary (http://www.bris.ac.uk/alspac/researchers/data-access/data-dictionary).

### Data analysis

After presenting descriptive data, we report bivariate associations between *COMT* rs4680 and prenatal risk and child outcomes. Analyses testing our primary hypothesis focus on the prenatal assessment of maternal anxiety at 32 weeks; maternal postnatal anxiety at 8 weeks and 33 or 97 months are included as controls in order to examine if there is a particular effect of maternal anxiety in pregnancy using linear regression models. For analyses of child working memory at age 8 years, we include total IQ as a control for general cognitive ability. All regression analyses account for obstetric, demographic, psychosocial covariates. We report unadjusted p-values and p-values corrected for multiple testing (x3 tests) based on the number of main study outcomes (see S1 File). After reporting the main analyses for maternal prenatal anxiety at 32 weeks gestation, we report supplementary analyses to examine if our predicitve models are robust to a series of confounds associated with gene-environment analyses. We tested if our results were robust to potential heteroskedacity using a seriers of robust joint tests implemented in PLINK [19]. Likewise, we implemented the approach of Keller [38] to eliminate covariate × environment or covariate × gene interactions as potential confounds in our analytical models. We then carry out a series of analyses to examine the specificity of the interaction between maternal mood and child genotype to predict child outcome. We test if 1) there are prenatal developmental timing effects (using the prenatal maternal anxiety assessment at 18 weeks gestation); 2) if the genetic moderation is specific to *in utero* exposure (by including moderation analyses of postnatal maternal anxiety and prenatal paternal anxiety); 3) if the prenatal anxiety effects are particular to anxiety or extend to maternal depression and if moderation effects are specific to genetic variation in *COMT* or extend to genetic variation in *BDNF*. For the latter analysis we focus on two SNPs in *BDNF* (rs11030121 and rs7124442) shown to moderate the effects of maternal prenatal anxiety on child *internalizing* symptoms.

Consistent with general practice, we did not include a missing data strategy where genetic data were not available; consequently, our sample size varies as a function of genetic data availability. The ALSPAC cohort is largely composed of Caucasian (>90%) participants. Given the small number of non-Caucasian participants, and potential influence of ethnicity on genotype frequencies, we focused our genetic analyses exclusively on Caucasians.

### Replication analyses

#### Sample

The PREDO cohort includes 4777 women recruited to the study between 2005 and 2009 at their first antenatal visits [39]. The final replication sample was comprised 425 mothers and their children with data available on maternal prenatal mood, child rs4680 genotype and symptoms of ADHD. The mothers participating in our study did not differ from other PREDO cohort mothers in their anxiety symptoms at any assessment point during pregnancy (all p-values ≥.13) and the participating children did not differ from other PREDO children in their rs4680 genotype or ADHD symptoms (all p-values ≥.10). The PREDO study protocol was approved by ethical committees of the Helsinki and Uusimaa Hospital District.

#### Measures

Maternal mood: Maternal anxiety symptoms were assessed with Beck Anxiety Inventory (BAI) at 32 weeks of gestation, 2 weeks postpartum, and 1.9–6.1 years after pregnancy. The BAI has excellent psychometric properties and good internal consistency (cronbach’s α = 0.92) [40].

COMT rs4680 genotype: Child genotyping was performed with Illumina Human Omni Express Exome 1.2. array. Rs4680 was directly genotyped and showed a call rate of 1.0, no deviation from Hardy-Weinberg equilibrium (p = .85), and minor allele frequency of 0.47 (G:G n = 92, G:A n = 214, A:A n = 119).

Child ADHD symptoms: Child ADHD symptoms over the past month were evaluated using the Conners’ 10-item scale [41]. Conners’ ADHD scale scores were square-root transformed to attain normality.

## Results

Table 1 provides descriptive data for measures of maternal mood and child neurodevelopmental outcomes according to child *COMT* (rs4680) genotypes. Attrition analyses indicated significant group differences in sociodemographic factors between participants providing data on maternal prenatal anxiety and child rs4680 and the remainder of the ALSPAC cohort. Participants included in our analysis were born to women who were older (t = -16.495, p<0.001), more educated (Χ^2^ = 195.529, p < .001) and who reported less household crowding (Χ^2^ = 267.216, p < .001) than the remainder of the ALSPAC cohort. Although significant (due to the large sample size), these group differences represented small effects (all Cohen’s d <0.29); these covariates were included in all analytical models described below to account for potential bias.

Child total IQ was associated with rs4680 in a univariate analysis (F_(2, 5022)_ = 3.44, p = 0.03). Post-hoc analyses indicated that children homozygous for the met allele showed higher total IQ compared to both other groups (Fischer’s least significant difference met/met vs val/met p = 0.04, met/met vs val/val p = 0.01, respectively); effect sizes can be estimated from results in Table 1. However, rs4680 was not significantly associated with child (r = 0.002, p = 0.881) or adolescent ADHD symptoms (r = 0.031, p = 0.074), or backward digit span test at age 8 years (r = -0.006, p = 0.690).

Child rs4680 genotype was not significantly associated with measures of pre- or postnatal maternal mood (r = -0.014–0.009, all p≥0.295) implying no confound of gene-environment correlation; neither was maternal prenatal anxiety or depression (at 18 or 32 weeks gestation) significantly associated with maternal rs4680 genotype (all p>0.222). Furthermore, for each child outcome there was no significant two-way interaction between child gender and maternal prenatal anxiety (all p ≥ 0.244) or between child gender and child rs4680 genotype (all p ≥ 0.441). Likewise we did not observe a significant three-way interaction (maternal prenatal anxiety x child gender x child rs4680) in the prediction of the main study outcomes (all p ≥ 0.097); therefore, males and females were combined and child gender was included as a covariate in all analyses.

### Child rs4680, maternal prenatal anxiety and working memory at age 8 years

**Fig 1:**
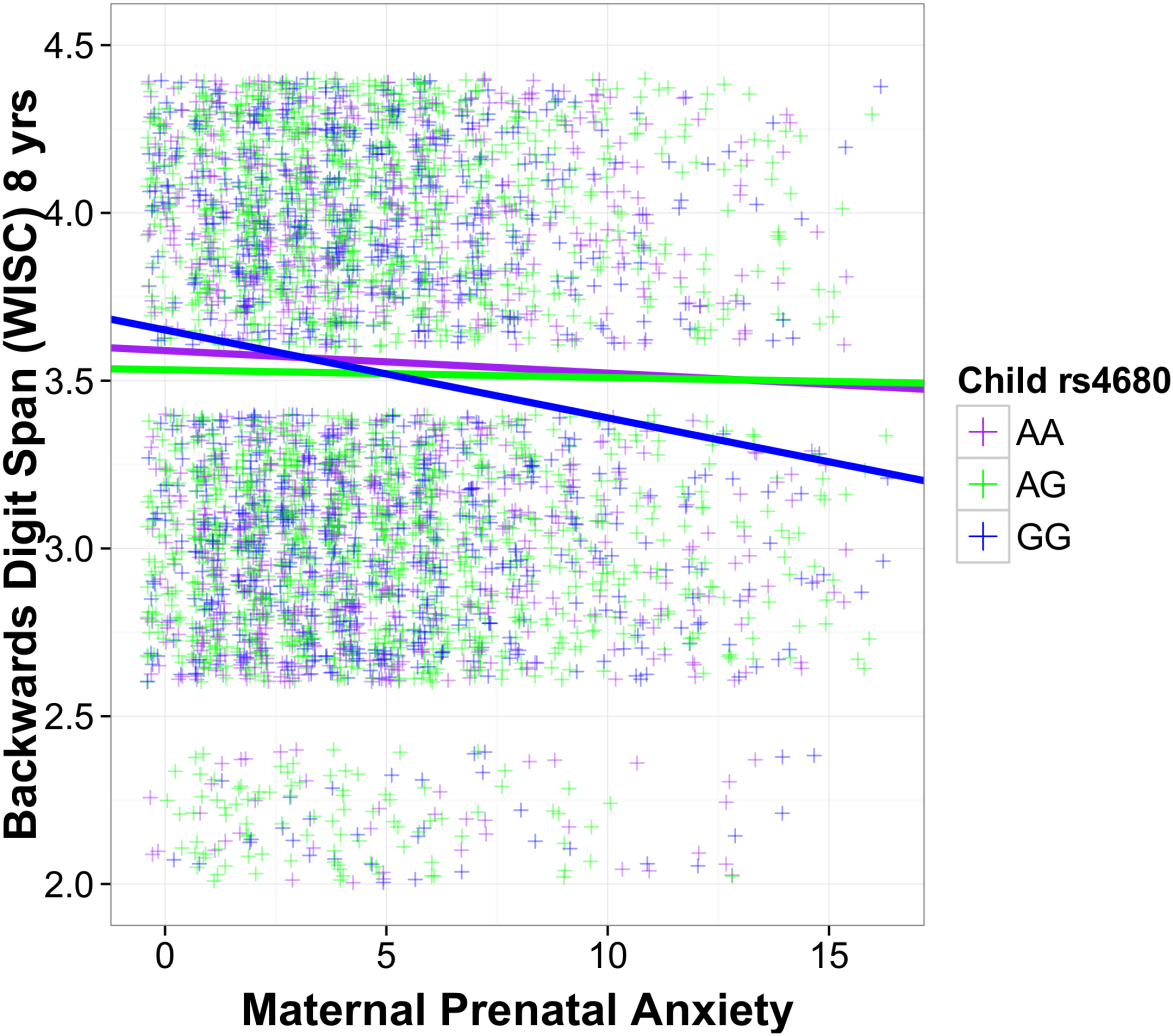
Maternal prenatal anxiety, child rs4680 genotype and working memory at 8 years of age. Maternal ratings of prenatal anxiety at 32 weeks of pregnancy (x axis) were plotted against child working memory (backward digit span) assessed as part of the WISC = Wechsler Intelligence Scale for Children at 8 years of age. Regression lines represent each of the three rs4680 genotypes: val/val (GG:blue), val/met (AG: green) and met/met(AA: purple). yrs = years.

A regression model which examined the main effects of our genetic and environmental predictors accounted for a significant proportion of the variance in child backwards digit span (R^2^ = 0.030, p<0.001). We also observed a significant interaction between maternal prenatal anxiety at 32 weeks and child rs4680 in predicting backward digit span at age 8 years: children with the val/val alleles exhibited a significantly shorter backward digit span with increasing prenatal maternal anxiety, after covarying a range of psychosocial and obstetric measures (Fig 1). The addition of the interaction term (maternal prenatal anxiey X child rs4680) increased the percentage variance explained in our model of child backwards digit span by 0.2%. These results were largely unchanged following adjustment for multiple testing (see S1 File). This effect was specific to backward digit span and did not extend to forward digit span or total IQ (data not shown; available from the authors); furthermore, the strength of this interaction was unchanged when child total IQ was covaried in the regression model (see S1 File).

### Child COMT rs4680 genotype, maternal prenatal anxiety and ADHD symptoms from age 4 to 15 years

Prenatal maternal anxiety at 32 weeks gestation predicted child ADHD symptoms at 4 years of age (B = 0.087, SE = 0.021, β = 0.127, p<0.001), independent of a number of covariates, including child rs4680 (see Table 2 and S1 File). This regression model accounted for approximately 9% of the variance in child outcome (R^2^ = 0.094, p<0.001). Prenatal maternal anxiety at 32 weeks gestation remained a significant predictor of the likelihood of ADHD at 15 years of age (B = 0.039, SE = 0.009, β = 0.190, p<0.001) in a regression model that accounted for approximately 5% of the variance in outcome (R^2^ = 0.046, p<0.001). The persistence of maternal prenatal anxiety predicting ADHD symptoms from 4 to 15 years was found despite modest stability in ADHD symptoms between these assessments (r = 0.246, p<0.001). The prediction from prenatal maternal anxiety was significantly moderated by child rs4680 genotype for ADHD symptoms at 4 and 15 years. The addition of the interaction terms (maternal prenatal anxiety X child rs4680) to our models increased the percentage variance explained in child and adolescent outcomes by 0.1% and 0.5%, respectively. After correction for multiple testing carriers of the val/val genotype exposed to higher levels of maternal prenatal anxiety showed higher symptoms of ADHD at age 4 year relative to val/met heterozygotes (adjusted p = 0.033) but not met/met homozygotes (adjusted p = 0.120).

**Table 2:** Maternal prenatal anxiety interacts with child rs4680 to predict symptoms of ADHD and working memory.

	SDQ (ADHD) 4yrs	DAWBA (ADHD) 15yrs	WISC (Backward Digit Span) 8yrs
^§^**Model 1**	β	β	β
Prenatal anxiety (32 wks)	0.06**	0.07**	0.01
Postnatal anxiety (8 wks)	0.05**	0.02	-0.03
Maternal anxiety (33 mths)	0.07***	-	-
Maternal anxiety (8 yrs)	-	0.10***	0.01
*COMT* rs4680			
val/val (reference)	-	-	-
val/met	-0.02	-0.03	>-0.01
met/met	-0.01	-0.08**	<0.01
^§^**Model 2 (Interaction analysis)**			
Prenatal anxiety *val/val	-	-	-
Prenatal anxiety *val/met	-0.09*	-0.15**	0.10**
Prenatal anxiety *met/met	-0.07*	-0.14**	0.10**

Each column represents results from independent regression models. Standardized regression coefficients (β) are presented for main effects models (model 1) and regression models containing both main effects and an interaction term between maternal prenatal anxiety at 32 weeks gestation and child rs4680 genotype (model 2).

^§^Both models include as covariates parenting behavior, maternal age at time of pregnancy, smoking and substance abuse during pregnancy, socioeconomic indicators: maternal education and household crowding, obstetric outcomes: birth weight, gestational age and child gender; see S1 File for full model output. ADHD = attention deficit and hyperactivity disorder; DAWBA = Development and Well-being Assessment, WISC = Wechsler Intelligence Scale for Children.

*≤0.05, **≤0.01, ***≤0.001 unadjusted p-values. wks = weeks, mths = months, yrs = years.

**Fig 2:**
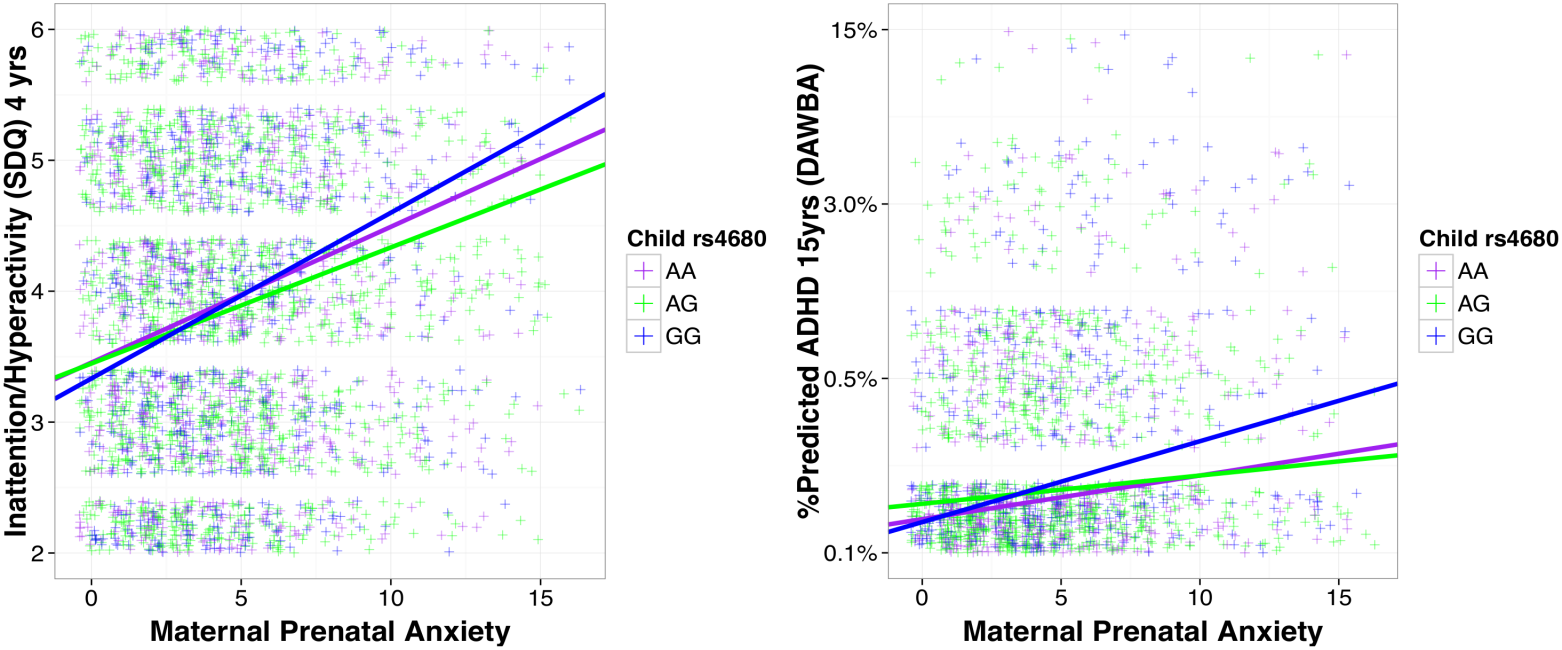
Maternal prenatal anxiety, child rs4680 genotype and symptoms of ADHD at 4 and 15 years of age. Maternal ratings of prenatal anxiety at 32 weeks of pregnancy (x axes) were plotted against child symptoms of ADHD from the Strengths and Difficulties Questionnaire (SDQ) at age 4 (left panel) and the likelihood of ADHD computed from the Development and Well-Being Aseessment (DAWBA) at age 15 years (right panel). Regression lines represent each of the three rs4680 genotypes: val/val (GG:blue), val/met (AG: green) and met/met(AA: purple). ADHD = attention deficit and hyperactivity disorder. yrs = years.

Likewise, carriers of the val/val genotype exposed to higher levels of maternal prenatal anxiety showed a greater likelihood of ADHD at age 15 years relative to both met/met (p = 0.009) and val/met genotypes (adjusted p = 0.006) (see Fig 2 and see S1 File).

### Replication analyses

In the PREDO sample maternal prenatal anxiety predicted child symptoms of ADHD at 3.5years (B = 0.225, SE = 0.103, β = 0.227, p = 0.030) independent of a number of covariates, including child rs4680. This regression model accounted for 17.1% of the variance in child outcome (R^2^ = 0.171, p<0.001). Likewise, maternal prenatal anxiety interacted with child rs4680 genotype to predict elevated symptoms of ADHD at 3.5years (B = -0.234, SE = 0.113, β = 0.170, p = 0.040), increasing the percentage variance explained in child outcome by 0.9% (R^2^ = 0.180, p<0.001). Analyses stratified by genotype demonstrated that the effects of maternal prenatal anxiety on child symptoms of ADHD were most pronounced in carriers of the val/val genotype (B = 0.226, SE = 0.142, β = 0.242, p = 0.065) relative to both val/met (B = 0.070, SE = 0.079, β = 0.076, p = 0.378) and met/met groups (B = 0.156, SE = 0.109, β = 0.152, p = 0.153).

### Supplementary analyses

Several sets of analyses were conducted to examine the robustness and specificity of the prenatal anxiety X child *COMT* genotype interaction in the prediction of markers of neurodevelopment. First, we tested if our results were inflated by heteroskedascity within our data (see [19]). We found significant variance inflation in child ADHD symptoms scores at age 4 and 15 years as a function of maternal prenatal anxiety (see S1, S2 and S3 Figs). The interaction between maternal prenatal anxiety and child rs4680 predicted child working memory (robust joint test corrected p = 0.019) and ADHD symptoms at age 15 years (robust joint test corrected p<0.01) using models robust to heteroscedasticity, but the robust joint test indicated that the interaction between maternal prenatal anxiety and child rs4680 was reduced (corrected p = 0.16). The reliability of the prenatal maternal anxiety X child *COMT* genotype was also suggested when we applied an analytic approach suggested by Keller [38]. This approach requires the inclusion of all two-way interactions between the genetic predictor (*G*: rs4680), the environmental factor (E: maternal prenatal anxiety) and all other covariates (*C*) within a linear model. The inclusion of all *G* x *C* and *E* x *C* terms did not substantively change our results for comparisons between val/val and met/met homozygotes. For example, carriers of the val/val genotype exposed to higher levels of maternal prenatal anxiety had shorter backward digit span (p = 0.019) and significantly more symptoms of ADHD at 4 (p = 0.048) and 15 years of age (p = 0.024) relative to the met/met group.

Next, we examined if maternal prenatal anxiety interacted with *maternal* rs4680 genotype to predict child outcome. It did not. The interaction term between maternal prenatal anxiety and maternal rs4680 did not predict child working memory (interaction terms all p>0.495) or child ADHD symptoms at 4 (interaction terms all p>0.333) or 15 years (interaction terms all p>0.565), and including the interaction by maternal *COMT* genotype did not substantively alter the interaction effect for child *COMT* genotype. Next, on the subset on whom paternal data in pregnancy were available, there was not reliable evidence for a genetic moderation of the prenatal paternal anxiety effect for working memory or inattention and hyperactivity at ages 4 and 15 years of age (details available from the authors).

Likewise, maternal prenatal anxiety at 32 weeks was the only measure of maternal mood in the pre or postnatal period to significantly interact with child COMT genotype to predict all of the three neurodevelopmental outcomes examined in this study, i.e., there was not a *post*natal maternal anxiety X child *COMT* genotype interaction (see S1 File). We then examined if the genetic moderation of the prenatal maternal anxiety effect extended to prenatal maternal depression at 18 or 32 weeks. The effects were similar, but somewhat weaker (see S2 File).

Finally, we considered *BDNF* polymorphisms as an alternative genetic moderator, given its known role in neurodevelopment and a prior finding from this sample suggesting that *BDNF* moderated the association between prenatal anxiety and internalizing symptoms [36]. There was no evidence that *BDNF* SNPs (rs11030121 and rs7124442) moderated the association between maternal prenatal anxiety and child neurodevelopmental outcomes (details available from first author).

## Discussion

By capitalizing on 15 years of data from a large community sample and an independent replication sample, we extended the research base on the prenatal developmental programming hypothesis by demonstrating a genetic moderation of the prenatal prediction of key markers of neurodevelopment in children and adolescence, and identifying catecholamine neurotransmission as one mechanism by which prenatal maternal anxiety may alter child neurodevelopment. The magnitude of the moderation effects was small, consistent with documented genotype X environment interactions so far reported for neurodevelopmental outcomes; nonetheless, the strengths of the study and the robustness of the prediction substantiate the persisting effect implied by the programming hypothesis for child and adolescent neurodevelopment.

To date, the mechanistic focus in research on prenatal maternal anxiety, depression and stress and its effect on child development is the HPA axis; that derives from substantial evidence from the experimental animal work [42]. Although there is now substantial evidence for a prenatal maternal anxiety effect on several aspects of child behavioral and biological development, confirmatory evidence for an HPA axis-linked mechanism is limited. That may reflect significant limitations in identifying causal mechanisms in non-experimental human work; alternatively, it may be that the mechanistic focus has been too narrow. Our finding that the prenatal maternal anxiety effect is moderated by child rs4680 that regulates COMT, which is involved in the inactivation of catecholamine neurotransmitters (dopamine, epinephrine, norepinephrine), implies that a broader biological model for understanding prenatal maternal anxiety effects is needed. Dopamine neurotransmission in the prefrontal cortex plays a major role in mediating neurodevelopment, including executive function [43] and symptoms of inattention and hyperactivity—although the latter remains a matter of some controversy [44]. Our findings implicating *COMT* for regulating the prenatal maternal anxiety prediction of executive function and inattention/hyperactivity are of interest for identifying a potential mechanism for work on prenatal anxiety and for expanding COMT-focused research on developing neurocognitive functions. It is particularly notable that our analyses of more than ten years’ of data from this large community sample is consistent with prior work on *COMT* moderating a prenatal anxiety exposure based on smaller and/or younger samples [16, 17]. Findings in ALSPAC and PREDO differ, at least in part, from those of Thompson et al., [17] who report stronger effects of maternal perinatal perceived stress on a broad-band measure of child behavioral and emotional problems in children homozygous for the met allele. It is not clear how to interpret the differences in findings, however, given that >40% of the children in the Thompson et al. study were born small for gestational age and their outcome measure incorporated a wide array of behaviors.

A developmental programming pattern is suggested by the finding on a persisting association between prenatal maternal anxiety and child outcomes through mid-adolescence [4, 45]. It remains to be seen if this effect extends into adulthood, which would further substantiate a “programming” effect (although see [9, 10]). It is also notable that the prenatal maternal anxiety prediction and genetic moderation were specific to working memory, rather than general IQ, and that this prediction was unchanged after accounting for general cognitive ability. That is notable given the particular interest in working memory and executive functions more generally in relation to *COMT* and several neuropsychiatric disorders, such as ADHD, for example.

How maternal anxiety in pregnancy may alter dopamine-linked brain development in the child is unclear; several different kinds of effects are possible. Genes associated with regulation of monoamines, such as *COMT*, are associated with the cortisol stress response [46], not ruling out an HPA axis-mediated effect. *COMT* expression in the fetal dorsolateral prefrontal cortex (DLPFC) increases across pregnancy (S4 Fig)(see [47]), with recent work showing prenatal enrichment of specific *COMT* transcripts within the developing brain (see [48]). The transcriptional profile of *COMT* within the DLPFC contrasts with that of *BDNF*, which is expressed at low levels in the fetal DLPFC. Future work is required to determine if these developmental differences in gene expression may underlie our significant *COMT* G x E findings and the absence of a *BDNF* G x E effect on child working memory and symptoms of ADHD. Prenatal epigenetic regulation of *COMT* expression is also possible. Ursini et al. report genotype-dependent associations between lifetime stress, DNA methylation of *COMT* and working memory [49]. Moreover, the authors describe a positive correlation between DNA methylation at the rs4680 locus and working memory and find lower DNA methylation in individuals homozygous for the rs4680 val allele exposed greater lifetime stress. Further work is required to determine if maternal prenatal anxiety influences the epigenetic regulation of *COMT* expression.

Several limitations of the study deserve mention. Although our prenatal maternal anxiety and child *COMT* genotype interaction results are compatible with prior studies (assessing children of different ages and using alternative phenotypes in some cases), an independent sample with all outcome measures assessed (e.g., symptoms of ADHD from 4–15 years and working memory) was not available for replication analyses. However, we do show that our findings can be replicated, at least in part, in an independent sample using similar constructs but different measures. Second, we relied on maternal reports for measures of prenatal anxiety and child symptoms of ADHD at 3.5 (PREDO), 4 and 15 years (ALSPAC), introducing a potential reporter bias. However, the interaction effect was also found for in-person testing of backward digit span in the ALSPAC cohort, which would not be subject to reporter bias. This study would also have been benefited from multi-informant reports of child/adolescent symptoms (e.g., paternal and teacher), which increase the sensitivity of symptom checklists for detecting psychiatric diagnoses [50]. Third, targeted candidate gene approaches have their limitations, although we have demonstrated that our findings on ADHD symptoms at age 15 and working memory are robust and not confounded by heteroskedasticity which can inflate gene-environment associations [19]. Finally, as with virtually all other demonstrations of GxE effects, the clinical implications of our findings may be limited given the small genetic moderation effect, which accounted for less than 1% of the variance in all outcomes assessed. Studies that use a genome-wide approach will be required to resolve the full extent, and clinical relevance, of genetic moderation in the context of maternal prenatal mood and child outcome. However, such studies will be methodologically challenging and complicated by the potential specificity of GxE effects, as observed in the current study (and see [36]). In this context, it should be noted the gene-environment effect size we report is somewhat comparable to main-effect estimates derived from recent large-scale polygenic analyses of ADHD [51].

The limitations of our study are offset by several strengths, including the longitudinal prospective design from pregnancy through the child’s adolescence, comparatively large sample size, extensive assessments that included in-person testing, detailed data that allowed us to examine the developmental specificity of this gene-environment effect and evidence of replication in an independent sample.

Further research that capitalizes on the experimental leverage of prenatal intervention is needed to extend these findings; further work is also needed on developmental changes in *COMT* effects, as a primary predictor and as a moderator of the prenatal anxiety effect, given age-based changes in COMT-related imaging effects that have been reported [52].

## Supporting information

S1 File Full regression models of maternal anxiety, child rs4680 and child/adolescent outcomes. (XLSX)

S2 File Full regression models of maternal depression, child rs4680 and child/adolescent outcomes. (XLSX)

S1 Fig Maternal prenatal anxiety and variance in child symptoms of ADHD at age 4 years. (PDF)

S2 Fig Maternal prenatal anxiety and variance in child working memory at age 8 years. (PDF)

S3 Fig Maternal prenatal anxiety and variance in symptoms of ADHD at 15 years of age. (PDF)

S4 Fig *COMT* expression in the dorsolateral prefrontal cortex across development. (PDF)
