# Supplementary material for: Maternal prenatal anxiety and child COMT genotype predict working memory and symptoms of ADHD
Source: PLoS One. 2017 Jun 14;12(6):e0177506. doi: 10.1371/journal.pone.0177506 (PMC5470664; doi:10.1371/journal.pone.0177506)

**S1 Fig:** Maternal Prenatal Anxiety and Variance in Child Symptoms of ADHD at age 4 years. Violin and box plots show the distribution of child inattention/hyperactivity symptoms from the Strengths and Difficulties (SDQ) questionnaire (y-axis) grouped by quartiles of maternal prenatal anxiety at 32 weeks (x-axis). Variance estimates are provided (blue text). Barlett's test for equality of variance shows significant differences across groups.

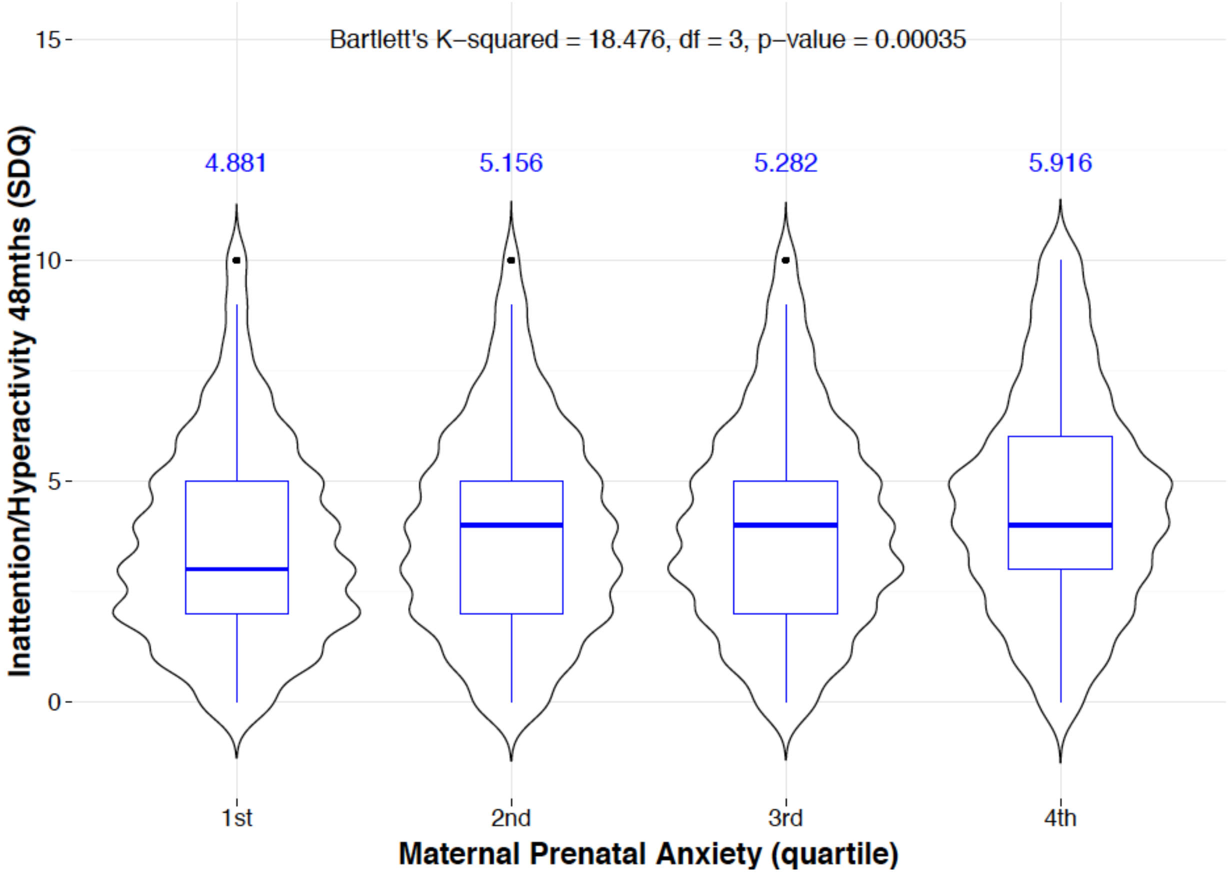

Supplement: S1 Fig — (PDF) [file pone.0177506.s003.pdf]
