# Supplementary material for: Maternal prenatal anxiety and child COMT genotype predict working memory and symptoms of ADHD
Source: PLoS One. 2017 Jun 14;12(6):e0177506. doi: 10.1371/journal.pone.0177506 (PMC5470664; doi:10.1371/journal.pone.0177506)

S3 Fig: Maternal Prenatal Anxiety and Variance in Symptoms of ADHD at age 15 years. Violin and box plots show the distribution of predicted likelihood of ADHD computed from the Development and Well-Being Assessment (DAWB, y-axis) grouped by quartiles of maternal prenatal anxiety at 32 weeks (x-axis). Variance estimates are provided (blue text). Barlett's test for equality of variance shows significant differences across groups.

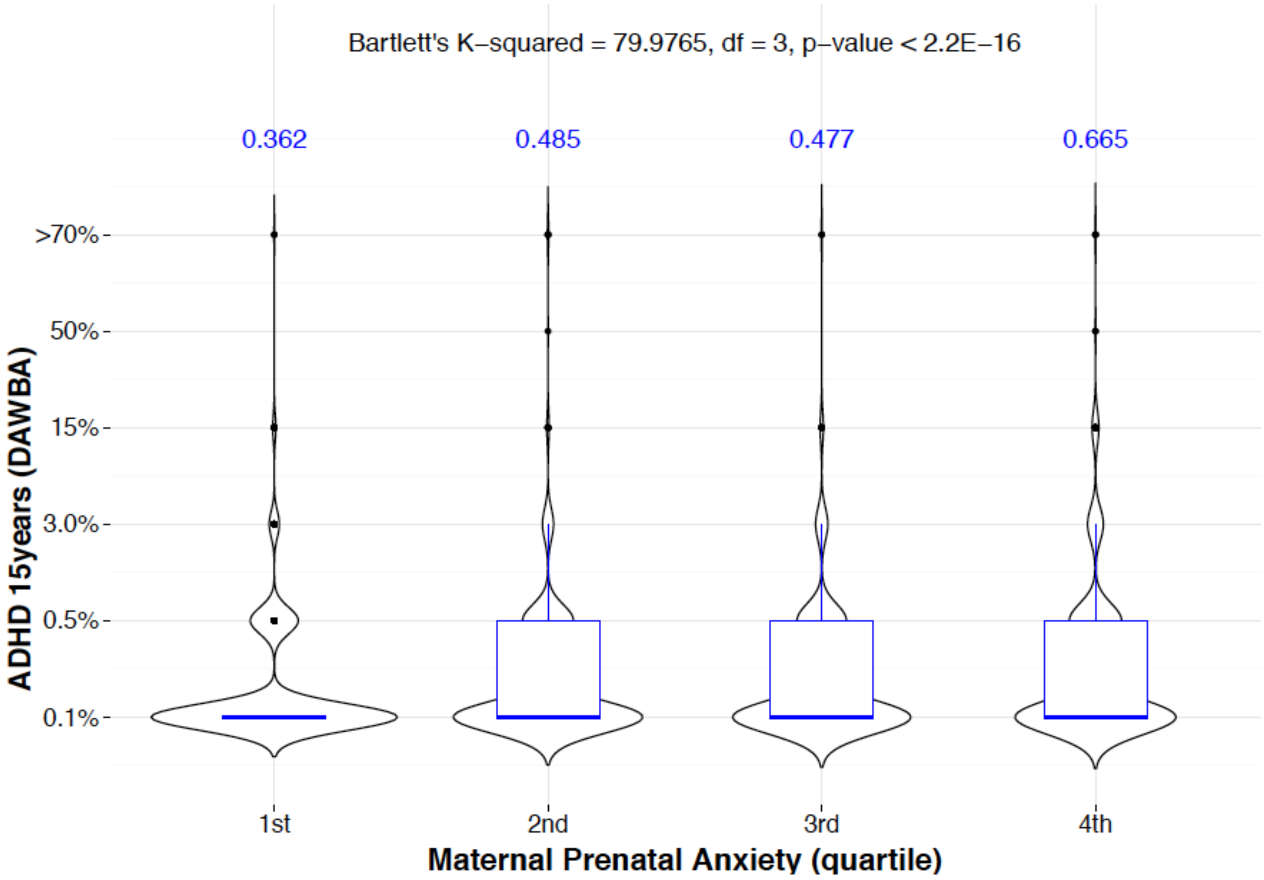

Supplement: S3 Fig — (PDF) [file pone.0177506.s005.pdf]
