# Supplementary material for: Maternal prenatal anxiety and child COMT genotype predict working memory and symptoms of ADHD
Source: PLoS One. 2017 Jun 14;12(6):e0177506. doi: 10.1371/journal.pone.0177506 (PMC5470664; doi:10.1371/journal.pone.0177506)

S4 Fig: *COMT* expression in the Dorsolateral Prefrontal Cortex Across Development. *COMT* expression (y-axis) derived from RNAseq analysis of human post-mortem dorsolateral prefrontal cortex collected from 8 to 456 weeks post conception (Age_PCW, x-axis). Red-dashed line represents 40 weeks post-conception. Data were sourced from http://www.brainspan.org.

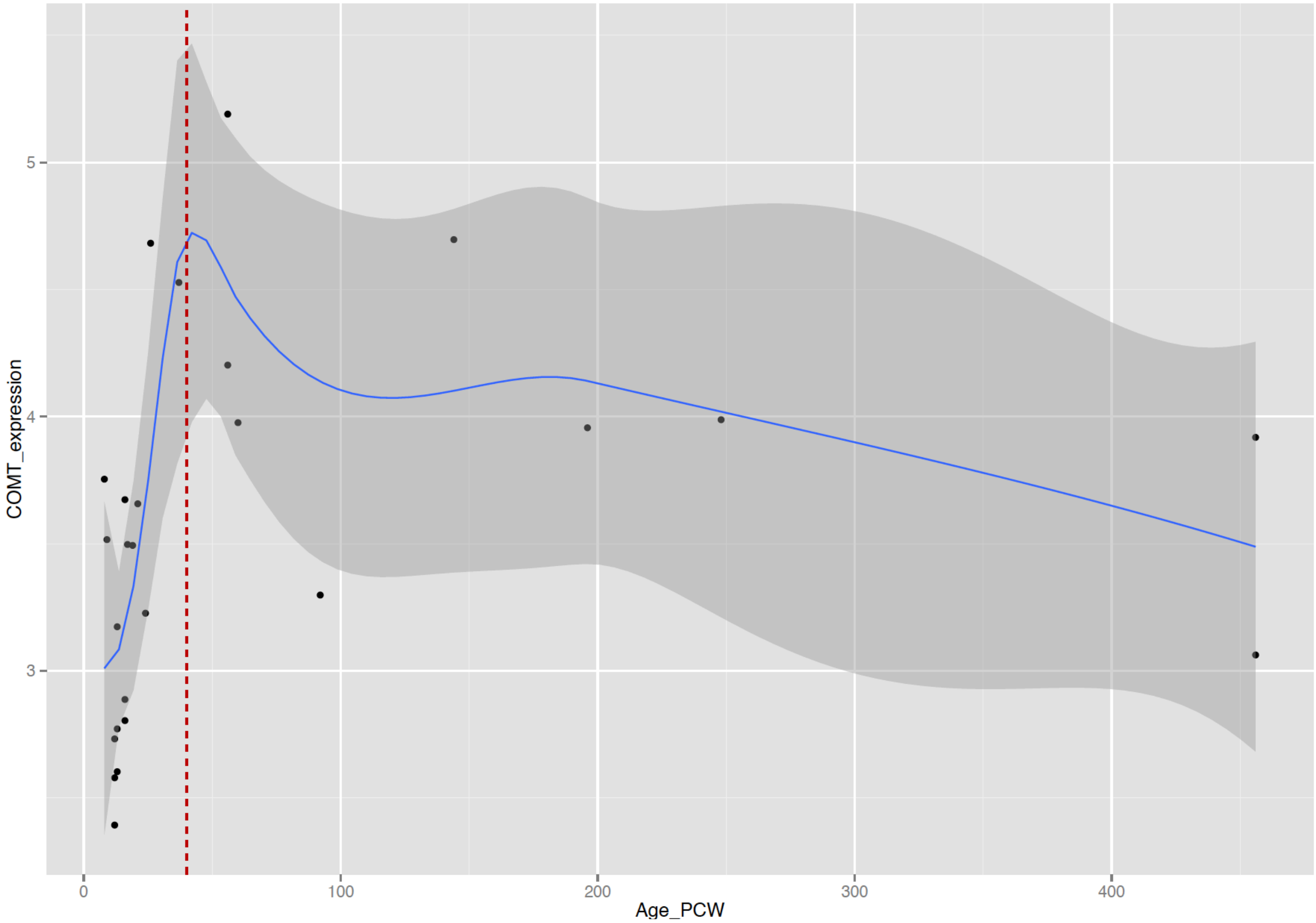

Supplement: S4 Fig — (PDF) [file pone.0177506.s006.pdf]
